# SuMO-Fil: Supervised multi-omic filtering prior to performing network analysis

**DOI:** 10.1371/journal.pone.0255579

**Published:** 2021-08-03

**Authors:** Lorin M. Towle-Miller, Jeffrey C. Miecznikowski, Fan Zhang, David L. Tritchler

**Affiliations:** 1 Department of Biostatistics, University at Buffalo, Buffalo, NY, United States of America; 2 Biostatistics Division, University of Toronto, Toronto, Ontario, Canada; University of North Texas, UNITED STATES

## Abstract

Multi-omic analyses that integrate many high-dimensional datasets often present significant deficiencies in statistical power and require time consuming computations to execute the analytical methods. We present SuMO-Fil to remedy against these issues which is a pre-processing method for Supervised Multi-Omic Filtering that removes variables or features considered to be irrelevant noise. SuMO-Fil is intended to be performed prior to downstream analyses that detect supervised gene networks in sparse settings. We accomplish this by implementing variable filters based on low similarity across the datasets in conjunction with low similarity with the outcome. This approach can improve accuracy, as well as reduce run times for a variety of computationally expensive downstream analyses. This method has applications in a setting where the downstream analysis may include sparse canonical correlation analysis. Filtering methods specifically for cluster and network analysis are introduced and compared by simulating modular networks with known statistical properties. The SuMO-Fil method performs favorably by eliminating non-network features while maintaining important biological signal under a variety of different signal settings as compared to popular filtering techniques based on low means or low variances. We show that the speed and accuracy of methods such as supervised sparse canonical correlation are increased after using SuMO-Fil, thus greatly improving the scalability of these approaches.

## Introduction

As the costs of high-throughput experiments continue to decrease [1], it is common to assay a variety of genomic information from large cohorts of patients at the level of single nucleotide variants (SNVs) [2], gene expression level via ribonucleic acid sequencing (RNA-Seq) [3] and, perhaps, at the metabolomic and proteomic levels [4]. In this setting, many sets of data are obtained where each set of variables is considered high-dimensional as it is obtained in a high-throughput setting. Analysis often proceeds with a combination of pathway, or *network*, methods that identify features across different ‘omic’ datasets that relate to each other and an outcome of interest [5]. It is believed that these integrated approaches of incorporating multiple layers of ‘omic’ information mimic Systems Biology and motivates the necessity for network analysis methods. It should be noted that oftentimes the terms “pathways” and “networks” are used interchangeably. For example, gene regulatory networks (GRNs) comprise of subsets of features within a single ‘omic’ dataset that relate to each other [6], but Creixell et al. [7] make a clear distinction between the two terms where “pathways” refer to within dataset relationships and “networks” refer to between dataset relationships. The rest of this paper will utilize these distinctions where network analysis will refer to integrated analyses aimed at identifying features spanning across multiple ‘omic’ datasets.

Certain complex diseases or clinical outcomes may be better understood through networks that span across multiple types of ‘omic’ information, and these networks can elucidate Systems Biology knowledge regarding the delicate interplay between DNA, DNA methylation, RNA, etc. For example, Danussi et al. [8] note a particularly interesting network by integrating copy number variation, gene expression, and gene mutation data obtained from The Cancer Genome Atlas (TCGA) project [9] where they discovered aggressive glioblastoma (GBM) associated with an amplified *RHPN2* gene that triggered undesirable molecular events. A multitude of other network detection studies [10, 11] have resulted from the TCGA project, which has supported the genomic data collection on approximately 11,000 patients across about 30 different types of tumors. In these studies, numerous data types were obtained through RNA-seq, MicroRNA sequencing, DNA sequencing, SNP detection, DNA methylation sequencing, and protein expression [9]. Most data types collected from TCGA studies and many other similar studies contain a large amount of features per ‘omic’ data type for thousands of subjects. In joint-level analyses, the dimensionality that results from the integration of multiple high-dimensional feature sets often produces heavily underpowered situations when attempting to adjust for multiple testing. To address this, many network detection algorithms that extend beyond just one type of data have raised the complexity of the analysis, which ultimately introduces limitations due to computational concerns [12]. To avoid the complexity of integrating the multi-omic data, some approaches analyze each ‘omic’ dataset separately to identify pathways within an individual ‘omic’ type and then later identify associated features from other ‘omic’ datasets, as done in Multi-Omic inTagrative Analysis (MOTA) [13].

A challenge with data of this magnitude involves the computationally expensive modeling of the biological networks that can consume massive amounts of central processing unit (CPU) time, particularly for re-sampling based procedures such as bagging, boosting, bootstrapping or permutation which are used in various methods such as the penalized canonical correlation analysis (PCCA) [14], the supervised sparse canonical correlation analysis (SCCA) [15], the supervised penalized canonical correlation analysis method [16], and the Decomposition of Network Summary Matrix via Instability (DNSMI) [17]. PCCA, supervised SCCA, DNSMI, and many similar methods impose lasso-type constraints on each dimension (e.g., each ‘omic’ data type) to produce sparse solutions, and tuning multiple lasso-type hyperparameters exponentiates the tuning process. For example, DNSMI implements an extended version of the Stability Approach to Regularization Selection (StARS) [18] that estimates stability for a grid set of two different lasso-type hyperparameters (e.g., one for each data type) and performs DNSMI on many subsamples of the data to obtain an optimally stable hyperparameter set. Tuning on stability has shown to produce more sparse solutions with reduced type I error as compared to popular techniques such as cross validation [18], but when incorporating into higher order problems, the subsampling schemes drastically increase the computation required. Many researchers using these network analyses currently have to find creative ways to remove features due to memory constraints, such as removing genes with unknown location followed by averaging values across adjacent genes [15] or removing features with low variance [16]. It should be noted however that removing features due to low variance and/or low means may sometimes be done when the biological interpretation suggests they contribute little information and not due to computational concerns, as done by Meng et al. [19] in their application to bladder cancer data obtained from TCGA.

Feature filtering techniques in analyses with only one ‘omic’ type have been previously explored to address underpowered feature selection after multiple testing adjustments. Bourgon et al. [20] demonstrate that filtering features using techniques that are independent of the underlying test statistics ultimately increased detection power, with favorability in filtering based on feature variance. Hackstadt and Hess [21] also showed that power was increased in the underlying analysis after removing features with low variance. Zhang et al. [22] proposed a feature filtering technique motivated by single ‘omic’ pathway analyses, called NARROMI, that filters features based on mutual information. Additionally, Tritchler et al. [23] compared various filtering techniques intended for clustering or single ‘omic’ pathway analysis and found that filtering based on summed values from the covariance matrix (SUMCOV) performed favorably with less noise in the underlying clustering results.

Although filtering and dimensionality reduction has been studied extensively for single-omic analyses, little has been explored to handle filtering in multi-omic studies. The authors Meng et al. [24] summarize dimensionality reduction techniques for integrated multi-omic data, but the methods presented include variations of CCA and extensions of principal component analysis (PCA) which too closely align with the downstream analyses and are not intended for simple pre-processing to reduce the number of features. The computational conundrum resulting from multi-omic network detection methods further exacerbates the need for relevant pre-processing filtering techniques as technological improvements allow for deeper assaying of ‘omic’ information and larger cohorts of patients are achieved.

Our novel approach addresses this weakness by providing a scalable dimensionality reduction technique for filtering or removing genes/features that are considered to be “irrelevant” (e.g., features that are not involved in the network) from the customary downstream supervised network analysis approaches. Advantages of this approach include its simple interpretation and improvement in speed of the downstream network analysis algorithms such as supervised SCCA. Another advantage is improved accuracy. That is, the biological network will be more easily discerned if the analysis reduces the amount of irrelevant or unimportant genes. In summary, specificity in final results may be improved by limiting the number of noisy features in the data and ultimately in the network selections themselves.

This work extends the filtering technique proposed by Tritchler et al. [23] to accommodate multiple ‘omic’ types for supervised network analyses by filtering features based on similarity measures across combinations of the data types and outcome of interest. We first introduce the Supervised Multi-omic Filtering (SuMO-Fil) algorithm, we then evaluate the performance of the algorithm under various simulations, and we apply the algorithm to a real world example. Our algorithm can be accessed freely in an R package on Github at https://github.com/lorinmil/SuMOFil. Functions are available to filter features using SuMO-Fil and perform simulations of a network system.

## Materials and methods

Our pre-processing feature filtering method, SuMO-Fil, applies to network analyses that include two data types and result in a continuous outcome, although simple extensions may be incorporated to accommodate higher dimensional problems. It is expected that a biological network will have features within one data type that are related to features within another data type, which together result in the outcome. For example, suppose a small set of highly methylated DNA locations leads to changes in gene expression patterns which ultimately result in a particular disease outcome. This network may be thought of as a causal chain with the outcome of interest, where the DNA methylation is the first data type and gene expression is the second intermediary data type. Furthermore, any features in a given data type that are independent of all features in the other data type and/or independent of the outcome should not be included in the network. More formally, this may be expressed as follows:

Let ***X*** = {*x*_1_, *x*_2_, …, *x*_*p*′_} and ***G*** = {*g*_1_, *g*_2_, …, *g*_*q*′_} denote the set of variables within datasets X and G that belong to a supervised network with outcome *y*. Letting *x*_*j*_ denote some variable in X and *g*_*k*_ denote some variable in G,

1. If *x*_*j*_⊥*g*_*k*_, then *cov*(*x*_*j*_, *g*_*k*_) = 0, *x*_*j*_ ∉ ***X***, and *g*_*k*_ ∉ ***G***.
2. If *x*_*j*_⊥*y*, then *cov*(*x*_*j*_, *y*) = 0 and *x*_*j*_ ∉ ***X***.
3. If *g*_*k*_⊥*y*, then *cov*(*g*_*k*_, *y*) = 0 and *g*_*k*_ ∉ ***G***.

**Fig 1:**
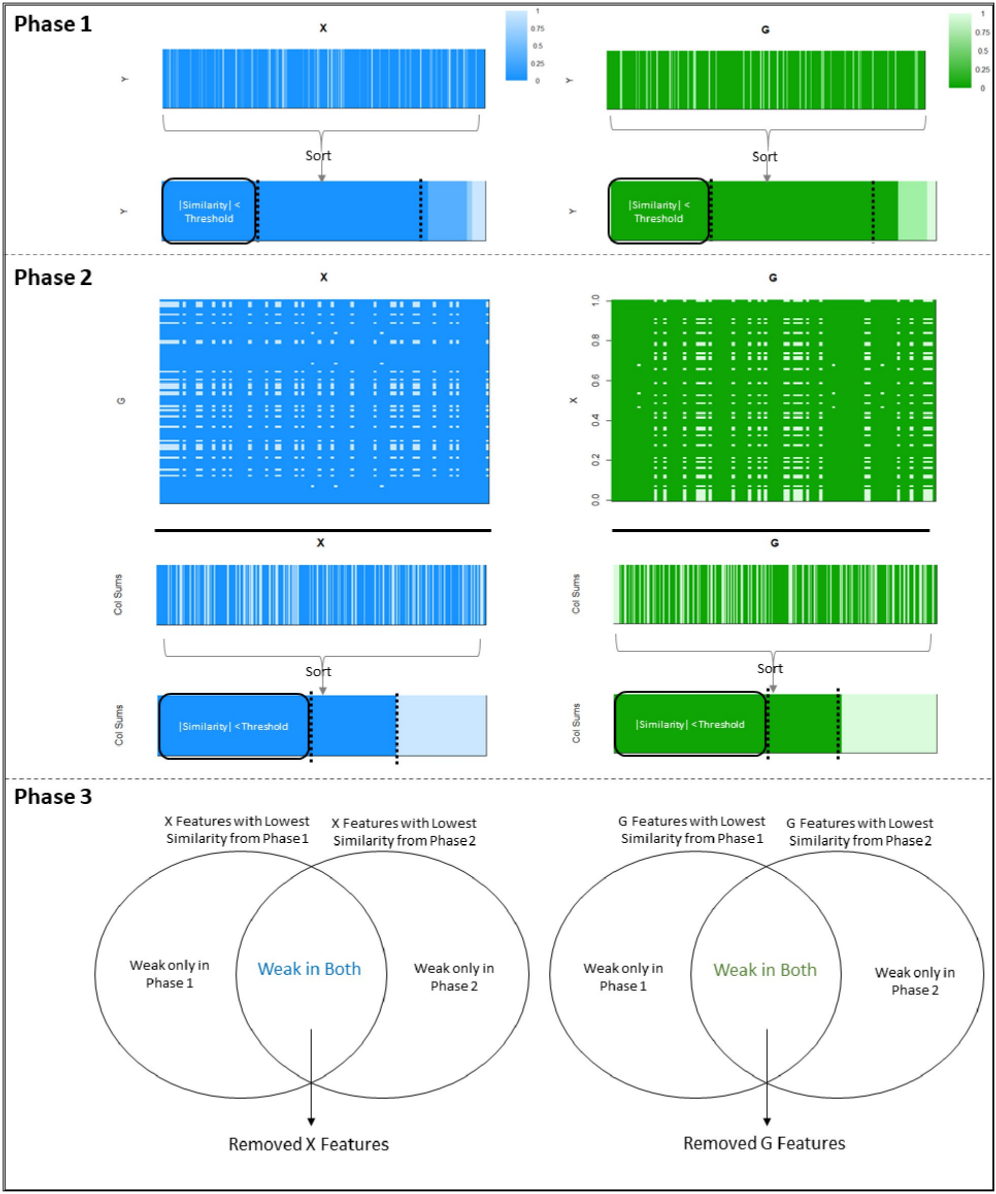
Filtering algorithm diagram. The filtering algorithm is performed in three phases. The first phase identifies features within the data types that have weak similarity with the outcome, the second phase identifies features that are weakly similar between the data types by summing the absolute value of similarities, and the third phase performs the final filtering step by removing any features that were identified as weak from phase 1 and phase 2. If features are weakly related to the outcome and weakly related with any features in the corresponding data type, then it is unlikely to be involved in the supervised network, and thus provides the motivation for SuMO-Fil. The phases are described in full detail in Algorithm 1.

With these network assumptions in mind, SuMO-Fil utilizes estimated similarity matrices (e.g., Pearson correlation) between the outcome and the data types. A thresholding criteria (e.g., features within the lowest k-mean similarity cluster) is then applied to the similarities, and filters are established based on features with the lowest similarities. This essentially removes any features in each data type that are not related to any features in the other data type or the outcome. The full description of the algorithm is detailed in Algorithm 1 and summarized in Fig 1.

**Fig 2:**
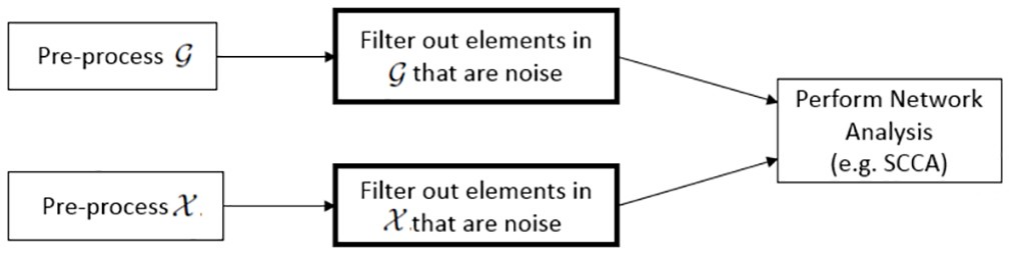
Suggested network analysis workflow. The proposed workflow when performing a supervised network analysis with a filtering step between initial pre-processing (e.g., normalization and quality control measures) and the primary analysis in order to improve results and reduce run times for downstream network detection methods.

It should be emphasized that the goals of SuMO-Fil are to remove irrelevant features (e.g., those features not involved in the network) within the data types prior to the downstream analysis and NOT to perform feature selection or network identification. Many network analysis methods assume that the number of features involved in the network are much smaller than the total number of features sequenced, an assumption known as *sparsity*, and the methods utilize constraints and techniques to accommodate this sparsity assumption. Feature selection would aim at selecting features that are significantly related to the outcome and would likely eliminate too many features, thus violating the sparsity assumption that is made in many network setting solutions. This filtering process is simply intended for improving the results and reducing computation time of downstream analyses and not to identify network features themselves. Fig 2 summarizes the intended workflow including the SuMO-Fil pre-processing step when performing network analysis.

It should be noted that popular, exploratory techniques such as the supervised SCCA [15] select network features by estimating weights for each feature and truncating non-network feature weights to zero. If a feature is either weakly correlated with the outcome or if the feature has little impact on maximizing correlation with the other data type, its weight will be set to zero. As a result, since SuMO-Fil removes features that are weakly correlated across datasets and weakly correlated with the outcome, they would likely have weights set to zero in the underlying supervised SCCA. When a weight in supervised SCCA is truncated to zero, it will be essentially ignored when maximizing the correlation across data types, thus implying that SuMO-Fil will not bias the underlying network results under similar methods. However, SuMO-Fil may not be appropriate if the downstream analysis includes hypothesis testing since it would potentially bias the results when incorporating correlation with the outcome as part of the filtering criteria.

### Notation

Suppose there is an observed continuous outcome **y** for *n* subjects. Additionally, suppose that each subject has two ‘omic’ data types collected, denoted as G and X. There are *q* total features for each subject within G and *p* total features for each subject within X, which results in G and X being *n* × *q* and *n* × *p*, respectively. Sub-matrices of sizes *n* × *q*′ and *n* × *p*′ are contained within G and X that make up the network features which explain the outcome, where there are *q*′ network features in data type G and *p*′ network features within data type X. In most network applications, a sparsity assumption is held, which would imply *q*′ << *q* and *p*′ << *p*.

### Filtering algorithm

Before the phases of the algorithm may be carried out, similarity matrices must first be estimated between G and X, G and **y**, and X and **y**. Once all similarity matrices are estimated, SuMO-Fil is then implemented. Note that the SuMO-Fil algorithm may accommodate any similarity measure where the smaller the absolute value corresponds to uncorrelated variables, and Pearson’s Correlation was used as the similarity measure for the simulations and data application in this manuscript.

The SuMO-Fil method comprises of three phases: Phase 1 gathers a list of features within both G and X to potentially be filtered based on low similarities with **y**, Phase 2 gathers a list of features within both G and X to potentially be filtered based on low similarities between G and X, and Phase 3 combines the results from Phases 1 and 2 to establish a final set of features to be filtered based on their intersecting low similarities. Phases 1 and 2 are performed separately, and Phase 3 will simply collect the intersecting results to construct a final set of features to be filtered. Note that filters on G and X are performed similarly and conducted independently, not conjointly. This will allow parallelization between the two data types in obtaining their set of features to be filtered.

In Phase 1, the absolute value of estimated similarities between **y** and all features within G will be captured (resulting in a vector of size *q*), and a thresholding criteria will be applied to the similarity vector (e.g., identify all features with a similarity less than a certain threshold). All G features that with an absolute similarity less than the threshold will be marked for potential filtration. A similar process would be repeated on all features within X to identify a set of features under this data type that are also weakly associated with the outcome **y**.

Phase 1 flags features for removal that are not related to the outcome, but does not address the association between data types. Hence, the goal of Phase 2 is to identify features within G that are not related to features within X, and similarly to detect features within X that do not appear to be related with features within G. The estimated similarity matrix between G and X is obtained and the absolute value of all estimated similarities is computed. For each feature of G within this absolute similarity matrix, we sum across all features of X (resulting in a vector of size *q*). All features of G with summed similarity less than a specified threshold should be flagged for potential filtering as those features do not show strong similarity with features of X. A similar process for X is conducted where the similarities across all G features are summed and features with values less than a specified threshold are flagged for potential removal.

Phase 3 combines Phases 1 and 2 by identifying the set of features in G and X that were flagged for potential filtration in both phases. Thus, by filtering out the intersecting features, we are removing features that are weakly similar to **y** and weakly similar between the distinct data types. By definition of the networks of interest, it is expected that the network features are related with the outcome and related to the other data type. Any features that violate both assumptions are likely features that will be removed by the SuMO-Fil method.

Establishing a thresholding criteria is not a trivial task, thus throughout this paper, we will estimate thresholding based on k-means clustering on the similarity vectors. More specifically, we will perform k-means clustering on a given similarity vector and identify all features contained in the cluster with the smallest cluster mean, resulting in a set of features with the lowest similarities. The appropriate number of clusters should be selected such that the cluster with the smallest mean adequately represents a set of features having low similarity with the outcome (suggesting that it would likely not be detected as a network feature in the subsequent network analysis). Additionally, the appropriate number of clusters should be small enough such that it selects a sufficient number of features in order to make an impact on either reducing the run times or limiting the number of false positives of the underlying analysis. The number of clusters used for phases 1 and 2 would not necessarily have to be equal and should be calibrated such that each phase identifies a reasonable amount of features for potential removal. All results in this manuscript produced reasonable results using 3 clusters for both phases, but this may need to be modified based on the distributions of the data. For example, if the similarities are heavily right skewed then a higher number of clusters may be needed in order to drill down to a smaller set of features for the smallest cluster.

### Simulations

Simulations were performed to verify the validity and consistency of the SuMO-Fil algorithm. The goals for the filtering algorithm are to retain all network features and maintain the sparsity assumption required for downstream methods while still removing enough features that would result in significant run time reductions and increased specificity. In addition to verifying the validity, SuMO-Fil will be compared against filtering based on low feature means as well as low feature variances, since they are popular approaches used in other studies [25–28]. The supervised SCCA method presented by Witten et al. [29] will be applied to the simulations before and after filtering, and changes in sensitivity and specificity will be compared.

We note many of the downstream network discovery techniques such as supervised SCCA are based on sparse solutions where many of the variables or features are assigned zero coefficients. These sparse approaches are reasonable as it is believed only a relatively small set of features are involved in these functional network approaches. Thus, we need to strike a balance to ensure that SuMO-Fil removes enough features to improve speed and accuracy of downstream methods, but does remove too much such that a sparse solution is no longer appropriate. One criteria to assure a sparse setting is to examine the measure *n*/*log*(*p*) [30] and ensure that *n*/*log*(*p*) is much larger than the total number of features within the active network(s), where *n* is the number of subjects and *p* is the total number of features within the data type. Since the total number of features within a network are not known in practical analyses, this measure is unobtainable for real data. However, for our simulations, the sparsity assumption is met when there is little change in the sparsity measure *n*/*log*(*p*) for both G and X when comparing before and after filtering.

**Algorithm 1** SuMO-Fil Algorithm

**Require**: $\lambda_{X_{1}}>0,\lambda_{X_{2}}>0,\lambda_{G_{1}}>0,\lambda_{G_{2}}>0,X,G,y$

 p=ncol(X)

 q=ncol(G)

 **for** *i* = 1 to *p* **do**

  *s*_*X*,*y*_[*i*] = |similarity (X[,i],y)|

 **end for**

 $phase1_{X}=\left\{i:s_{X,y}\left[i\right]<\lambda_{X_{1}}\right\}$

 **for** *i* = 1 to *p* **do**

  *s*_*X*,*G*_[*i*] = 0

  **for** *j* = 1 to *q* **do**

   *s*_*X*,*G*_[*i*] = *s*_*X*,*G*_[*i*] + |similarity (X[,i],G[,j])|

  **end for**

 **end for**

 $phase2_{X}=\left\{i:s_{X,G}\left[i\right]<\lambda_{X_{2}}\right\}$

 *phase*3_*X*_ = *phase*1_*X*_ ∩ *phase*2_*X*_

 **for** *j* = 1 to *q* **do**

  *s*_*G*,*y*_[*j*] = |similarity (G[,j],y)|

 **end for**

 $phase1_{G}=\left\{j:s_{G,y}\left[j\right]<\lambda_{G_{1}}\right\}$

 **for** *j* = 1 to *q* **do**

  *s*_*G*,*X*_[*j*] = 0

  **for** *i* = 1 to *p* **do**

   *s*_*G*,*X*_[*j*] = *s*_*G*,*X*_[*j*] + |similarity (G[,j],X[,i])|

  **end for**

 **end for**

$phase2_{G}=\left\{j:s_{G,X}\left[j\right]<\lambda_{G_{2}}\right\}$

 *phase*3_*G*_ = *phase*1_*G*_ ∩ *phase*2_*G*_

 **return** *phase*3_*X*_, *phase*3_*G*_

**Fig 3:**
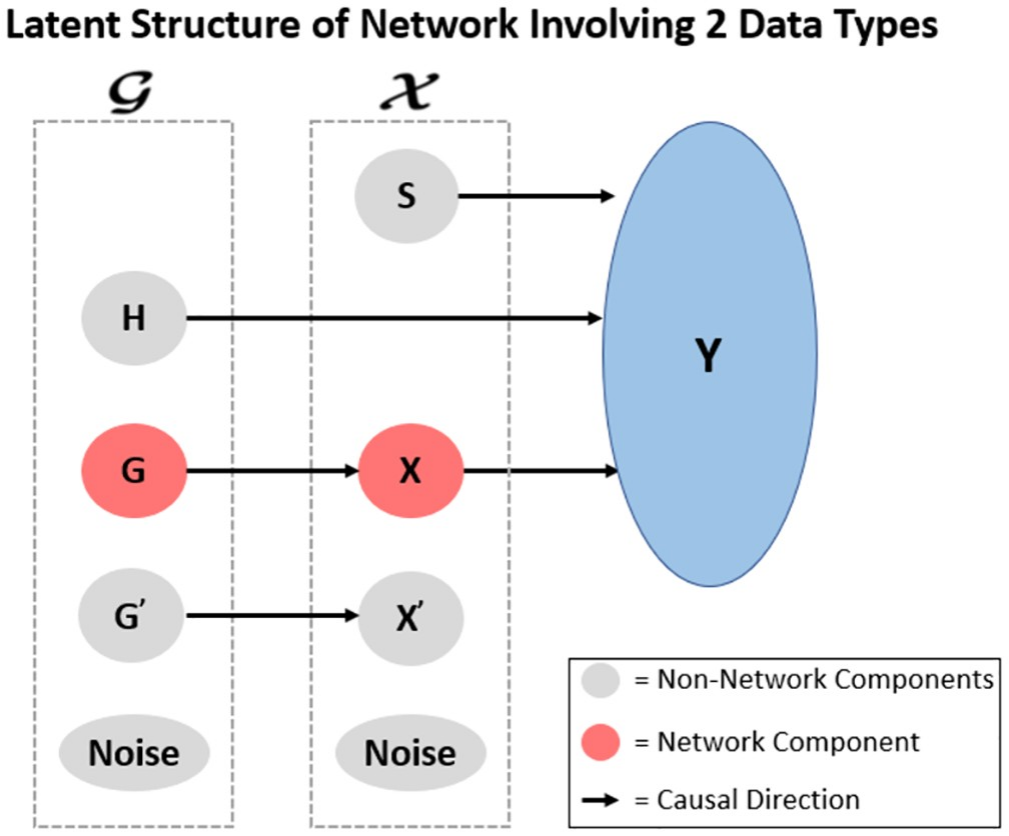
Diagram of latent variables in a network system with the outcome y. Each of the latent variables (**S**, **H**, **X**, **G**, **X**′, **G**′, and **Noise**) describe a subset of features within data types G and X with their corresponding relationships. **S** describes the set of features within X that are related to **y** but not any features in G; **H** describes the set of features within G that are related to **y** but not any features in X; **X** describes the network features within X which is related to the network features within G and **y** (note that there are p′ features in this subset); **G** describes the network features within G which is related to the network features within X and **y** (note that there are q′ features in this subset); **X**′ describes the set of features within X that are related to a set of features within G but not related to **y**; **G**′ describes the set of features within G that are related to a set of features within X but not related to **y**; and **Noise** describes the features within X and G that are not related to each other or **y**. The variables within the respective dotted boxes summarize the various components contained within each of the data types, and the arrows signify a relationship and imply correlation between the corresponding components. SuMO-Fil thus describes “irrelevant” features as those belonging to components **S**, **H**, **X**′, **G**′, and **Noise**. Note that this figure was adapted from [17].

For each simulation, we simulate matrices G and X with an outcome vector **y**. G and X are designed to contain network features which ultimately explain **y**. In addition to the network features, there may be other noise-like features within G and X. For example, there may be features in G and X that are individually related to **y** but not part of a network, as well as features in G and X that are related with each other but not with **y**. Additionally there may be features that are purely noise uncorrelated with the outcome and uncorrelated with all other features. Ultimately, Fig 3 shows the types of features and correlation relationships that are contained in G and X. As there are numerous types of relationships indicated in Fig 3, there will be numerous parameters to set in each simulation. In short, we construct a global covariance structure for the latent variables, where the simulation parameters help define the strength of the covariance values. Then, for each simulated “subject”, latent values are simulated based on a multi-variate normal distribution with zero mean and the global latent covariance structure. Finally, using the “subject’s” latent value as the mean and the global latent variance, feature values are simulated based on a normal distribution. Note that the simulations used in this paper mimic the network simulations used by Zhang et al. [17]. Further theoretical details on the simulations may be found in S1 Appendix.

#### Parameter selection

The parameters used in the mathematical models to generate the simulations were adjusted to consider various levels of signal strength and various quantities of features. The parameters for establishing weak, moderate, and strong network signal strength mimic the parameters used by Zhang et al. [17]. The same number of subjects were used in all simulations with a sample size of 1000. The signal strengths were each generated for a small, medium, and large number of total features (approximately 5000, 10000, and 20000 features within each data type, respectively). The number of features within the networks were kept small in order to accommodate the sparsity assumption and selected to reflect similar numbers described by Chin et al. [12]. Simulations were repeated 500 times under each setting to confirm reproducibility. As noted previously, thresholding was based on k-means throughout this paper, and *k* = 3 clusters were used under each phase for the SuMO-Fil method. Additionally, Pearson’s Correlation was used as the similarity measure for the SuMO-Fil method. To remain comparable in the number of filtered features, the competing methods of filtering based on low feature means and low variances was also based on thresholds set by k-means with *k*′ = 9 clusters. Note that *k*′ was set to 9 instead of 3 since SuMO-Fil is based on the intersection of low thresholds as opposed to the feature means and feature variances which filter based solely on one threshold. Refer to Table 1 for the details on the total number of features used for the simulations.

**Table 1:** Summary of simulations.

Item	Small	Medium	Large
Subjects	1000	1000	1000
Features in X	5165	15165	25165
Features in **S**	50	50	50
Features in **X**	15	15	15
Features in **X**′	100	100	100
Noise Features in X	5000	15000	25000
Features in G	5140	10140	20140
Features in **H**	30	30	30
Features in **G**	10	10	10
Features in **G**′	100	100	100
Noise Features in G	5000	10000	20000

The number of features across the simulations for the small, medium, and large number of features are summarized in the table. The same parameters for weak, moderate, and strong signal strengths were generated across each number of features for a total of 9 (3 × 3) different simulations, where each simulation contained 500 replications. The relationships between each of the components within X and G are described in Fig 3. In summary, **S** denotes the features within X that are related to the outcome but not related to features within G; **X** denotes the network features within X that are related to some features within G and related to the outcome; and **X**′ denotes the features within X that are related to some features within G but not related to the outcome. Similar relationships within G are described by **H**, **G**, and **G**′.

## Results

### Simulation results

The SuMO-Fil method, along with the competing filtering methods based on low feature means and low feature variances, were applied to each of the simulations under all parameter settings. For each simulation, we examined the change in *n*/*log*(*p*) before and after filtering and the sensitivity of what was filtered. The reduction in computation time for executing supervised SCCA, changes in sensitivity, and changes in specificity were also assessed on a subset of the simulations before and after each of the filtering methods.

Table 2 summarizes the average number of network features that were erroneously removed across all filtering techniques, along with the total number of features removed. Filtering based on low variances outperformed the other methods under weak network signal strength, but it performed poorly under moderate and strong network signal strengths. In fact, filtering by low variance often erroneously filtered all network features under the moderate and strong network signal strengths. The SuMO-Fil method outperformed filtering by low means under most simulation settings, with the exception for network features within X under the moderate network signal strength setting. Additionally, under all simulation settings, the SuMO-Fil method removed more total features than by filtering based on means or variances.

**Table 2:** Summary of simulation filtering results.

Simulation Settings			Total # Features Removed			Avg # Network Features Removed		
Signal	Total #	Data	Technique			Technique		
Strength	Features	Type	SuMO-Fil	Low Mean	Low Variance	SuMO-Fil	Low Mean	Low Variance
Weak	45305	X	3376	1091	904	0.34	0.76	0
Weak	45305	G	2704	876	720	0.22	0.53	0.00
Moderate	45305	X	3515	1315	165	0	0	15
Moderate	45305	G	2684	1062	135	0	0.28	9.2
Strong	45305	X	7339	1088	165	0	0	15
Strong	45305	G	6364	878	140	0	0.11	10
Weak	25305	X	2089	671	547	0.34	0.78	0
Weak	25305	G	1389	448	375	0.21	0.46	0
Moderate	25305	X	2245	820	165	0.01	0	15
Moderate	25305	G	1507	558	130	0.01	0.34	9.1
Strong	25305	X	5791	659	165	0	0	15
Strong	25305	G	3729	441	140	0	0.10	10
Weak	15305	X	841	232	196	0.40	0.81	0.01
Weak	15305	G	835	228	192	0.15	0.45	0
Moderate	15305	X	1278	280	162	0.02	0	14.7
Moderate	15305	G	1263	274	124	0.01	0.33	8.8
Strong	15305	X	2242	229	161	0	0	14.7
Strong	15305	G	2132	229	138	0	0.11	9.9

The total number of features removed and the number network features erroneously removed are averaged across 500 simulations under each setting accounting for various number of total features and network signal strengths. The simulations contained 15 network features within data type X and 10 network features within data type G. An ideal filtering technique would eliminate no network features to maintain signal for the primary analysis. Additionally, an ideal filtering technique would remove enough features to make an impact on run times for the primary analysis while maintaining sparsity assumptions. The results indicate that the variance filtering technique performs favorably for weak network signals, but performs poorly for the moderate and strong signal strengths in regards to both the number of features removed and the number of network features erroneously removed. The SuMO-Fil performs favorably compared to both the mean and variance filtering techniques under most simulation settings.

**Fig 4:**
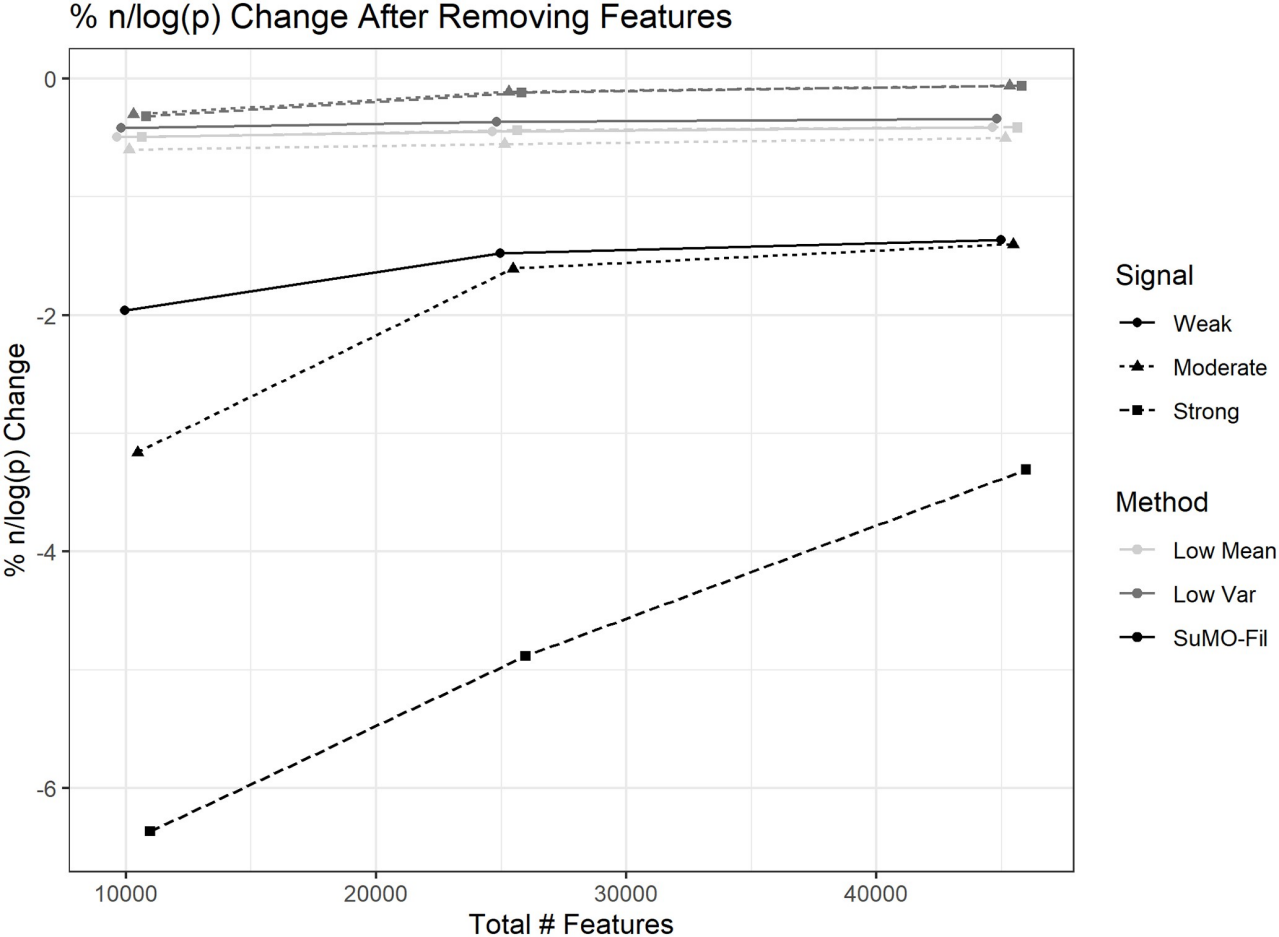
Percent change in sparsity. The average percent change in *n*/*log*(*p*) before and after removing features across the simulations from the nine different settings and the corresponding confidence interval bars. The points on each line correspond to small, medium, and large simulations from left to right. Also note that the total number of features is the number of features within both X and G combined, prior to any removing of features. Due to the high number of simulations, the confidence interval bars are very tight.

The SuMO-Fil method removed between 10 and 37 percent of features in both X and G under all simulation settings with an average of 5 percent change in *n*/*log*(*p*), as summarized in Fig 4. Since the changes in this sparsity measure were minimal, it suggests that the sparsity assumption has been maintained after filtration. The low mean and low variance filtering methods generated smaller changes to the *n*/*log*(*p*) measure as compared to the SuMO-Fil method since they did not remove as many features.

**Fig 5:**
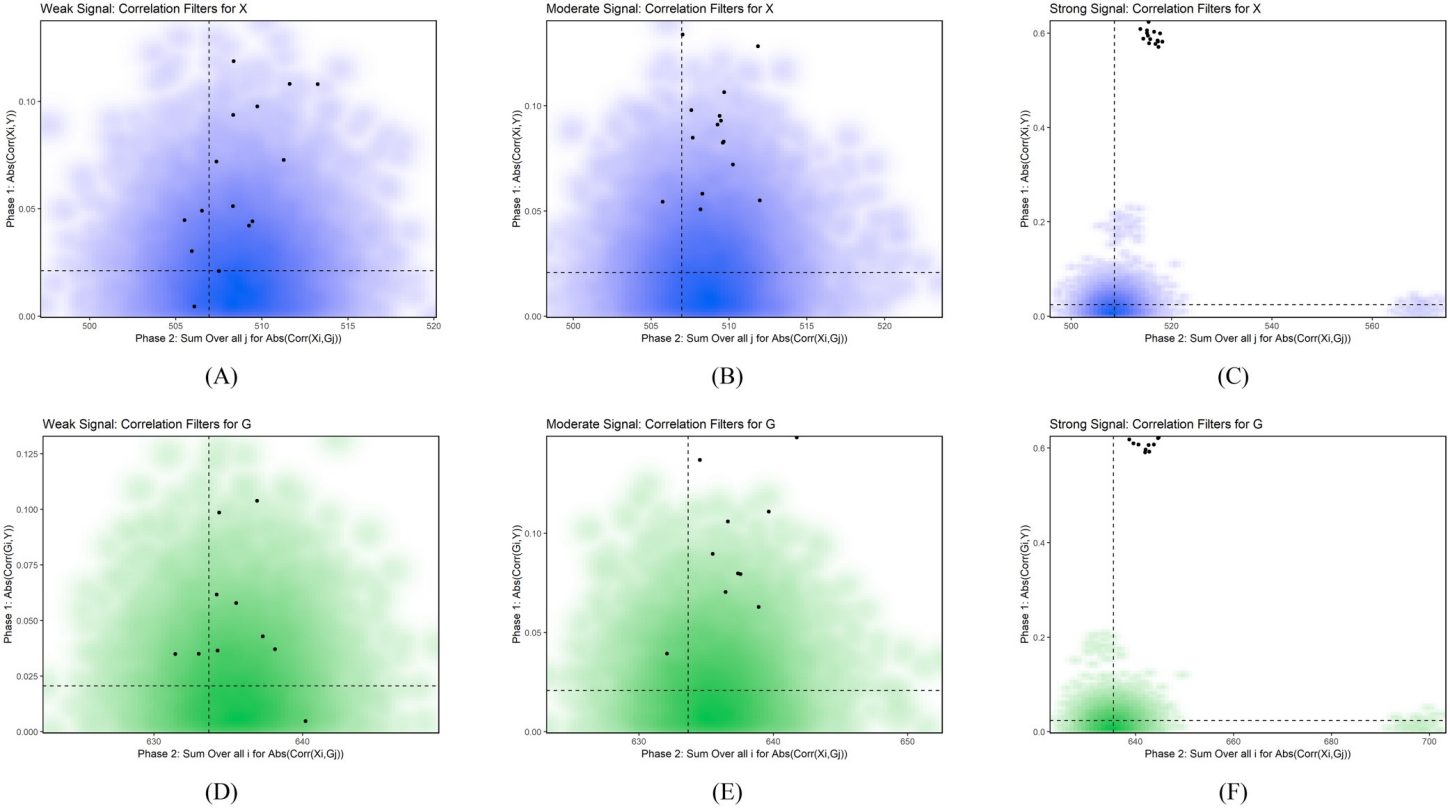
Representative simulations. This figure shows representative simulations for weak, moderate, and strong signal strength under the simulations with a large number of features (45305). The black points represent the features that belong to the network. The dashed lines within each plot represents the k-means cluster threshold obtained from the SuMO-Fil method, where all points within the bottom left quadrant of each plot represent the features that will be removed. Plots (A) and (D) correspond to the correlation measures for X and G, respectively, for a representative simulation under the weak signal setting; plots (B) and (E) correspond to the correlation measures for X and G, respectively, for a representative simulation under the moderate signal setting; plots (C) and (F) correspond to the correlation measures for X and G, respectively, for a representative simulation under the strong signal setting. In (A), we see 1 point (network feature) in the bottom left quadrant that would be erroneously removed from the SuMO-Fil method.

All simulations with strong signal did not remove any network features under the SuMO-Fil method, and the weak and moderate signal strength simulations erroneously removed less than one network feature on average from both X and G. Fig 5 displays representative simulations under the various signal strengths with a large number of features.

To assess the reduction in run times for downstream analysis due to the filters using SuMO-Fil, the supervised SCCA method [15] was employed on 64-bit Windows 10 with Intel Core i7–7700HQ, CPU of 2.80GHz, and 8.00 GB of RAM. Run times were collected for 5 simulations under each simulation setting. Table 3 summarizes the run times, changes in sensitivity, and changes in specificity of the network selections before and after filtering. SuMO-Fil took longer to execute than the other filtering methods, however, it resulted in the largest reduction in execution of supervised SCCA. Also, results were drastically improved in regards to sensitivity and specificity under most simulation settings after applying SuMO-Fil as opposed to performing the supervised SCCA either on the unfiltered data or the data with filters based on low means and low variance.

**Table 3:** Changes in network selections.

Simulation Settings		Technique	Sensitivity	Specificity	# Selected	Filtering+SCCA Run time (min)
Signal Strength	Total # Features					
Weak	45305	No Filter	0.32	0.993	*x* = 203, *g* = 103	31.7
Weak	45305	SuMO-Fil	0.80	0.996	*x* = 118, *g* = 100	14.4 + 28.8
Weak	45305	Low Means	0.04	0.995	*x* = 118, *g* = 94	<0.1 + 35.7
Weak	45305	Low Variance	0.20	0.994	*x* = 194, *g* = 95	0.3 + 39.2
Moderate	45305	No Filter	0.92	0.991	*x* = 237, *g* = 215	30.9
Moderate	45305	SuMO-Fil	0.92	0.992	*x* = 188, *g* = 190	15.1 + 31.1
Moderate	45305	Low Means	0.92	0.991	*x* = 219, *g* = 199	<0.1 + 33.9
Moderate	45305	Low Variance	0	0.989	*x* = 293, *g* = 226	0.2 + 37.2
Strong	45305	No Filter	1	0.972	*x* = 706, *g* = 576	32.3
Strong	45305	SuMO-Fil	1	0.980	*x* = 499, *g* = 409	15.8 + 21.8
Strong	45305	Low Means	1	0.973	*x* = 676, *g* = 551	0.1 + 38.7
Strong	45305	Low Variance	0	0.898	*x* = 2576, *g* = 2036	0.2 + 40.6
Weak	25305	No Filter	0	0.996	*x* = 72, *g* = 42	17.8
Weak	25305	SuMO-Fil	0	0.996	*x* = 62, *g* = 50	4.3 + 13.4
Weak	25305	Low Means	0	0.995	*x* = 70, *g* = 52	<0.1 + 15.6
Weak	25305	Low Variance	0	0.995	*x* = 72, *g* = 56	<0.1 + 16.0
Moderate	25305	No Filter	1	0.993	*x* = 114, *g* = 93	16.6
Moderate	25305	SuMO-Fil	0.96	0.994	*x* = 96, *g* = 74	4.3 + 13.0
Moderate	25305	Low Means	0.96	0.994	*x* = 107, *g* = 90	<0.1 + 15.7
Moderate	25305	Low Variance	0	0.967	*x* = 504, *g* = 336	<0.1 + 16.3
Strong	25305	No Filter	1	0.977	*x* = 356, *g* = 254	17.0
Strong	25305	SuMO-Fil	1	0.992	*x* = 117, *g* = 99	4.3 + 9.2
Strong	25305	Low Means	1	0.978	*x* = 329, *g* = 241	<0.1 + 16.1
Strong	25305	Low Variance	0	0.979	*x* = 315, *g* = 215	<0.1 + 16.4
Weak	15305	No Filter	0	0.996	*x* = 17, *g* = 19	6.5
Weak	15305	SuMO-Fil	0	0.997	*x* = 14, *g* = 17	0.7 + 5.0
Weak	15305	Low Means	0	0.997	*x* = 17, *g* = 18	<0.1 + 6.1
Weak	15305	Low Variance	0	0.997	*x* = 17, *g* = 18	<0.1 + 6.0
Moderate	15305	No Filter	0.920	0.999	*x* = 14, *g* = 23	6.6
Moderate	15305	SuMO-Fil	1	0.987	*x* = 64, *g* = 78	0.7 + 4.5
Moderate	15305	Low Means	0.92	0.981	*x* = 84, *g* = 107	<0.1 + 6.0
Moderate	15305	Low Variance	0.04	0.954	*x* = 242, *g* = 238	<0.1 + 6.2
Strong	15305	No Filter	0	0.997	*x* = 15, *g* = 19	6.7
Strong	15305	SuMO-Fil	0.720	1	*x* = 10, *g* = 9	0.7 + 3.6
Strong	15305	Low Means	0	0.998	*x* = 14, *g* = 12	<0.1 + 6.2
Strong	15305	Low Variance	0	0.861	*x* = 722, *g* = 709	<0.1 + 6.0

The median network results after applying supervised SCCA [15] to 5 simulations under each simulation setting. Supervised SCCA was applied to the simulations before filtering and after filtering based on SuMO-Fil, low means, and low variance. Under the simulations with 45305 total features, SuMO-Fil at minimum maintained sensitivity (while improving sensitivity under weak signal strength) and increased specificity by reducing the amount of noise in the network selections. SuMO-Fil also maintained or increased specificity under simulations with 25305 total features, although sensitivity was decreased under both SuMO-Fil and low mean filters with moderate signal strength. SuMO-Fil maintained or increased sensitivity under simulations with 15305 total features, although specificity was decreased under moderate network signal. Filtering based on low variance often decreased sensitivity to 0 while drastically increasing the number of noise selected and increasing run times. Performing supervised SCCA after SuMO-Fil produced the shortest run times under most simulation settings.

### Application

Uterine Corpus Endometrial Carcinoma (UCEC) is the fourth most common cancer amongst women in the United States with about 50,000 new cases reported and approximately 8000 deaths in 2013 alone [31]. Due to the extensive prior research of UCEC data and sufficiently large obtainable sample size, UCEC was selected as one of the tumor types assessed in the Pan-Cancer analysis project within the TCGA database where mutation, copy number, gene expression, DNA methylation, MicroRNA, and RPPA were collected from the tumor samples [32].

It has been shown from prior studies that the *p53* pathway is associated with many cancers, where *p53* can be indirectly inactivated when *MDM2* is overexpressed, *PTEN* or *INK4A/ARF* is mutated, the *Akt* pathway is deregulated, and other events occur [33]. We focus on chromosome 10, as it contains the *PTEN* gene and will utilize DNA methylation as one data type and gene expression as the other data type. Furthermore, prior research has shown that myometrial invasion is associated with tumor grade and patient survival [34], thus percent tumor invasion will be used as an outcome for this application. The percentage of positive lymph nodes has also been shown to relate to patient survival [35–37], so total pelvic lymph node ratio (LNR) will be used as a secondary outcome.

The data collected within TCGA for UCEC contained 587 subjects with 485577 DNA methylation features and 56830 gene expression features, and after focusing on chromosome 10 the data resulted in 24109 DNA methylation features and 2156 gene expression features. For each outcome, SuMO-Fil was applied using k-means for the thresholding with *k* = 3 clusters for each phase, and Pearson’s correlation was used for the similarity measure. The competing filtering methods involving low mean and low variance filtering were also applied using thresholds based on the sets of features in the lowest k-means cluster using *k* = 9 clusters. The supervised SCCA proposed by Witten and Tibshirani [15] was applied on the unfiltered data and on the data after each of the competing filtering techniques. Each feature was scaled to zero mean and unit variance prior to performing SuMO-Fil and supervised SCCA, but low mean and low variance filtering were performed on the unscaled data to maintain their natural interpretation.

Hamming distance captures the total number of discrepancies between two sets [38] and was calculated to compare the overlapping network node results across the different methods. The network’s node selections from either percent tumor invasion or pelvic LNR are summarized in S1 File, along with whether the features were removed by any of the filtering techniques.

#### Percent tumor invasion

After removing any subjects with either a missing/out of range (e.g., values not between 0 and 100) percent tumor invasion and any DNA methylation or gene expression features with less than 5 distinct values, the data resulted in 386 subjects with 18933 DNA methylation features and 2050 gene expression features. Table 4 summarizes the filtering and network selection results. SuMO-Fil is the only filtering method that did not remove any previously selected network features under the unfiltered data. In fact, both low mean and low variance filtering removed all of the previously selected gene expression features from the unfiltered data. This is likely attributed to the high number of features removed due to the highly right skewed feature means and variances which resulted in a large cluster to be removed. After applying SuMO-Fil, the supervised SCCA identified 3 new gene expression features that were previously missed in the unfiltered data. Of these includes *miR-4296* which has been shown to be related to cell death functions [39], as well as the *RF00019* gene which belongs to the *Y_RNA* family. *Y_RNA*’s are non-coding RNA’s, but they are believed to associate with endocrine-related cancers [40]. After applying low mean and low variance filtering, many more DNA methylation features were selected as compared to the unfiltered analysis, one of which (*cg02307823*) corresponds to the *PTEN* gene.

**Table 4:** Network selection summaries for percent tumor invasion.

Filtering Technique	# Features Filtered (# Selected in Unfiltered Network)	# Network Features Selected (# Selected in Unfiltered Network)	Filtering+SCCA Run time (mins)
Unfiltered		*M* = 290, *R* = 19	0 + 6.9
SuMO-Fil	*M* = 4225(0), *R* = 407(0)	*M* = 98(98), *R* = 5(2)	0.5 + 4.6
Low Means	*M* = 4643(27), *R* = 1579(19)	*M* = 1413(205), *R* = 45(0)	<0.1 + 4.1
Low Variance	*M* = 6902(56), *R* = 1862(19)	*M* = 1725(92), *R* = 28(0)	0.1 + 3.0

Supervised SCCA [15] was performed on the unfiltered data, after SuMO-Fil, after low mean filtering, and after low variance filtering. Run times and the number of features are summarized. Let *M* denote DNA methylation and *R* denote gene expression.

Table 5 displays the Hamming distances between the supervised SCCA results using the unfiltered data versus the filtered data. The supervised SCCA results are most comparable between the unfiltered data and the SuMO-Fil filtered data. The distances in network results after applying low mean or low variance filters are all high which is likely due to the much higher number of features selected. Interestingly, the Hamming distances are also high between results after applying low mean and low variance filtering, suggesting that they are selecting different high-dimensional sets of variables.

**Table 5:** Hamming distance for percent tumor invasion results.

	SuMO-Fil	Low Means	Low Variance
Unfiltered	*M* = 192, *R* = 20	*M* = 1293, *R* = 64	*M* = 1831, *R* = 47
SuMO-Fil		*M* = 1375, *R* = 50	*M* = 1763, *R* = 31
Low Means			*M* = 2094, *R* = 53

Supervised SCCA [15] was performed on the unfiltered data, after SuMO-Fil, after low mean filtering, and after low variance filtering. This table presents the Hamming distance which counts the number of discrepancies between network selections. The larger the Hamming distance suggests the selections are more different from each other. Let *M* denote DNA methylation and *R* denote gene expression.

#### Pelvic lymph node ratio

After removing subjects with a missing pelvic LNR and any DNA methylation or gene expression features with less than 5 non-zero values, there were 458 remaining subjects with 18901 DNA methylation features and 2066 gene expression features. Table 6 summarizes the number of features filtered by each method, the number of network features selected, and the run times. SuMO-Fil removed the least amount of features compared to low mean and low variance filtering, but it was the only method to retain all previously selected features under the unfiltered data. Since the feature means and variances were heavily right skewed, obtaining a set of low mean/variance features resulted in a large collection to be removed. SuMO-Fil reduced the number of selected features while maintaining a consistent subset from what was previously selected before filtering, and while low mean and low variance filtering reduced the number of features selected, the DNA methylation results were not as consistent with the unfiltered results. Although the low variance filtering removed many previously selected features, it selected two new gene expression features, *ANAPC16* and *TIMM23* which were not selected by any other method. The *TIMM23* gene has been shown to be downregulated for Endometriosis [41], and *ANAPC16* has potentially higher rates of alterations in certain cancers [42]. *PTEN* and *KLLN* genes are both associated with endometrial cancers [43], and SuMO-Fil retained one of the DNA methylation feature selections (*cg08859916*) corresponding to those genes that were also selected in the unfiltered data. However, there were 3 other *PTEN* DNA methylation features that were previously selected in the unfiltered data that were missed after applying SuMO-Fil (*cg02307823*, *cg06947206*, and *cg23149470*). Although low mean filtering removed 3 *PTEN* DNA methylation features that were previously selected in the unfiltered data, it did recover one DNA methylation feature (*cg09254926*) that was not discovered by any other method.

**Table 6:** Network selection summaries for pelvic lymph node ratio.

	# Features Filtered	# Network Features Selected	Filtering+SCCA
Filtering Technique	(# Selected in Unfiltered Network)	(# Selected in Unfiltered Network)	Run time (mins)
Unfiltered		*M* = 1607, *R* = 174	0+ 7.3
SuMO-Fil	*M* = 3554(0), *R* = 466(0)	*M* = 648(648), *R* = 79(79)	0.5+ 5.3
Low Means	*M* = 4668(226), *R* = 1594(105)	*M* = 548(59), *R* = 20(20)	<0.1+ 4.6
Low Variance	*M* = 6749(531), *R* = 1933(166)	*M* = 927(686), *R* = 9(7)	<0.1+ 3.3

Supervised SCCA [15] was performed on the unfiltered data, after SuMO-Fil, after low mean filtering, and after low variance filtering. Run times and the number of features are summarized. Let *M* denote DNA methylation and *R* denote gene expression.

Table 7 shows the Hamming distances between the network results under the different methods. Since the unfiltered data selected many more features than the filtered data, the Hamming distances are all high compared to the filtered results since Hamming distance counts the total number of discrepancies.

**Table 7:** Hamming distance for pelvic lymph node ratio results.

	SuMO-Fil	Low Means	Low Variance
Unfiltered	*M* = 959, *R* = 95	*M* = 2037, *R* = 154	*M* = 1162, *R* = 169
SuMO-Fil		*M* = 1148, *R* = 59	*M* = 657, *R* = 78
Low Means			*M* = 1409, *R* = 27

Supervised SCCA [15] was performed on the unfiltered data, after SuMO-Fil, after low mean filtering, and after low variance filtering. This table presents the Hamming distance which counts the number of discrepancies between network selections. The larger the Hamming distance suggests the selections are more different from each other. Let *M* denote DNA methylation and *R* denote gene expression.

## Discussion

Network analysis techniques such as DNSMI [17] and supervised SCCA [15] are critical for the understanding of large collections of datasets such as TCGA project. However, they can be either computationally intensive or select massive amounts of features and as such would greatly benefit from dimensionality reduction such as gene filtering. We note the SuMO-Fil approach improves computation times and accuracy while also maintaining a sparse setting required for most downstream penalization based network discovery algorithms. Additionally, we found that the SuMO-Fil approach outperforms popular filtering techniques used in single-omic studies based on maintaining signal. By reducing run times, more resources can be spent on the formal analysis while still maintaining consistent results and assumptions. Additionally, improved results may be achieved by limiting the amount of noise in the underlying data. To our knowledge, no pre-processing filtering methods have been published expressly for multi-omic data. We note that other software packages such as Sklearn in Python are very flexible and useful and may be adaptable for this purpose, however, SuMO-Fil has been customized to be mated in R which mates with algorithms such as SCCA and DNSMI [44].

Our simulations suggest that the SuMO-Fil method removes more features while maintaining sparsity and maintaining network signal as compared to the low mean and low variance filtering approaches. However, the thresholding criteria was based on k-means clustering to better match the thresholding criteria set for the SuMO-Fil method and additional thresholding parameters may be tuned for less conservative filtering. Although, despite the conservations set by the thresholding criteria, the competing choices still removed more network features on average. It should also be noted that the low variance feature filtering produced highly unstable results, thus further motivating a more robust filtering scheme for more complex multi-omic data. Real life examples also showed favorable results for the SuMO-Fil method because it largely maintained network signal discovered prior to filtering while unveiling novel genes previously undetected in the unfiltered data.

SuMO-Fil filters based solely on similarity, and any low variance features that are correlated with other features or the outcome will still survive the SuMO-Fil filters. Benchmarking SuMO-Fil assumed the downstream analysis utilized correlation between features and the outcome, but some research questions may involve differences in variance [45]. Additional work should explore SuMO-Fil’s performance and impact on variance-based downstream analyses. SuMO-Fil also does not consider within dataset correlation, but methods handling a single ‘omic’ type often identify modules of associated features based on the within dataset correlation, as done in a method presented by Miecznikowski et al. [46]. Although SuMO-Fil requires removed features to be weakly correlated with the outcome and weakly correlated with the corresponding data type, any method that utilizes within dataset correlation or similarities should be used with caution when using SuMO-Fil.

The second phase of SuMO-Fil takes the summation of the absolute similarity across all variables in the other dataset. This could potentially be diluted in situations where a feature is highly correlated with a small subset of features in the other dataset, and other summary measures should be considered. See S2 Appendix for results using median as opposed to summation for the second phase of SuMO-Fil. It should be noted that filtering based on means would be equivalent to filtering based on summation since the mean would just be the summation divided by the same constant for each feature.

There are several extensions of the SuMO-Fil method for consideration in future work. SuMO-Fil is currently limited to two data types and is currently not dynamic to handle additional data types. In a setting with more than two data types, one approach to consider would be to calculate similarity matrices between each of the data types and apply filters where the relationships are expected. This approach would become more complex as the number of data types increases and would require prior network relationship knowledge. We also caution that our approach should not be combined with downstream testing based approaches as our supervised approach will introduce a bias that could violate multiple testing corrections designed to control the Type I error.

The SuMO-Fil method also does not model for additional subject covariates that may be available for analysis. One *ad hoc* approach in this setting may be to compute residuals from a model involving the outcome and additional covariates. These residuals can then serve as the “outcome” in our filtering method. We are exploring this approach in a future manuscript. Additionally, SuMO-Fil requires an outcome of interest for the multi-omic filtering, and future work should explore multi-omic filtering for unsupervised settings. One possible solution could involve filtering based solely on the sum of the absolute correlations between the data types, as done in Phase 2 of SuMO-Fil.

We note that the estimate of run time reduction and changes in sensitivity/specificity in Table 3 are based on simulations of modest size and not the entire 500 simulations as employed for the other simulation estimates. This is due to the amount of resources required per deployment of the supervised SCCA procedure. In addition, our method was applied and tested in R version 3.4.4 [47]. Due to memory constraints in R, it cannot allocate extremely large matrices. This poses a problem when calculating correlation matrices where the number of features within G and X are large. To accommodate these constraints, the SuMO-Fil method may be applied on a row by row basis which significantly slows down run times for executing SuMO-Fil. Although the filtering method would only need to be applied once, other techniques and programming languages may be considered in obtaining the correlation matrices in order to avoid the memory limitations in R.

In summary, implementing SuMO-Fil as a pre-processing step prior to performing supervised network analyses may reduce run times while also reducing the amount of type I/type II errors in the network results.

## Supporting information

S1 Appendix Simulation details. (PDF)

S2 Appendix Alternative summary measures for SuMO-Fil. (PDF)

S1 File DNA methylation and gene expression network selections from UCEC application. (XLSX)
