# Supplementary material for: SuMO-Fil: Supervised multi-omic filtering prior to performing network analysis
Source: PLoS One. 2021 Aug 3;16(8):e0255579. doi: 10.1371/journal.pone.0255579 (PMC8330944; doi:10.1371/journal.pone.0255579)
Supplement: S1 Appendix — (PDF) [file pone.0255579.s001.pdf]

S1 Appendix

Simulation Details

Network systems described in this manuscript involve two data types, $\mathcal{G}$ and $\mathcal{X}$ with a continuous outcome **Y**. For our simulations, it is assumed that there are latent variables that describe sub-matrices contained in $\mathcal{G}$ and $\mathcal{X}$. It is assumed that there are latent variables that describe the sub-matrix of network features **G** contained in $\mathcal{G}$ that lead to the behavior of network features **X** contained in $\mathcal{X}$, which ultimately explain **Y**. There is also an interactive effect between **G** and **X**. In addition to the network features, there may be other features that comprise $\mathcal{G}$ and $\mathcal{X}$. There are latent variables that describe sub-matrices **S** and **H** within $\mathcal{G}$ and $\mathcal{X}$ that are individually related to **Y** but not each other. These relationships may be expressed as a system of linear equations shown in (1). The covariance matrix Σ' for the latent variables (**Y**, **X**, **G**, **S**, **H**)^{T} is defined in (2).

$$
\begin{aligned}
\mathbf{Y} &= \beta_{Y|X.G}\mathbf{X} + \beta_{Y|G.X}\mathbf{G} + \beta_{Y|S}\mathbf{S} + \beta_{Y|H}\mathbf{H} + \epsilon_{Y|X,G,S,H} \\
\mathbf{X} &= \beta_{X|G}\mathbf{G} + \epsilon_{X|G} \\
\mathbf{G} &= \epsilon_{G}
\end{aligned} \tag{1}
$$

$$
\Sigma' = \begin{bmatrix}
\sigma_{Y|X.G.S.H} + \sigma_{X|G}\beta^2_{Y|X.G} + \sigma_G\tau^2 + \sigma_S\beta^2_{Y|S} + \sigma_H\beta^2_{Y|H} & \sigma_{X|G}\beta_{Y|X.G} + \tau\sigma_G\beta_{X|G} & \sigma_G\tau & \beta_{Y|S}\sigma_S & \beta_{Y|H}\sigma_H \\
\sigma_{X|G}\beta_{Y|X.G} + \tau\sigma_G\beta_{X|G} & \beta^2_{X|G}\sigma_G + \sigma_{X|G} & \sigma_G\beta_{X|G} & 0 & 0 \\
\sigma_G\tau & \sigma_G\beta_{X|G} & \sigma_G & 0 & 0 \\
\beta_{Y|S}\sigma_S & 0 & 0 & \sigma_S & 0 \\
\beta_{Y|H}\sigma_H & 0 & 0 & 0 & \sigma_H
\end{bmatrix} \tag{2}
$$

There are also latent variables that describe sub-matrices $\mathbf{G}'$ and $\mathbf{X}'$ within $\mathcal{G}$ and $\mathcal{X}$ that are related

to each other but not related to $\mathbf{Y}$. Additionally, there are also sub-matrices $\mathbf{e_G}$ and $\mathbf{e_X}$ within $\mathcal{G}$ and $\mathcal{X}$ that contain independent noise variables. The covariance matrix Σ defined in (3) describes the relationships between all latent variables $(\mathbf{Y}, \mathbf{X}, \mathbf{G}, \mathbf{S}, \mathbf{H}, \mathbf{X}', \mathbf{G}', \mathbf{e_X}, \mathbf{e_G})^{\mathrm{T}}$.

$$\Sigma = \begin{bmatrix} \Sigma' & \mathbf{0} & \mathbf{0} & \mathbf{0} & \mathbf{0} \\ \mathbf{0} & \beta^2_{X|G}\sigma_G + \sigma_{X|G} & \sigma_G\beta_{X|G} & \mathbf{0} & \mathbf{0} \\ \mathbf{0} & \sigma_G\beta_{X|G} & \sigma_G & \mathbf{0} & \mathbf{0} \\ \mathbf{0} & \mathbf{0} & \mathbf{0} & \sigma_{e_X} & 0 \\ \mathbf{0} & \mathbf{0} & \mathbf{0} & 0 & \sigma_{e_G} \end{bmatrix} \tag{3}$$

All simulations performed were generated under a multivariate normal distribution with the covariance structure defined in (3). For a given "subject" i, the latent values are first randomly generated from $MVN(\mathbf{0}, \Sigma)$. Denote the generated latent values for subject i as $\mathbf{L}_i = (y_i, x_i, g_i, s_i, h_i, x'_i, g'_i, e_{X_i}, e_{G_i})^T$. The features for subject i are then completed by generating each sub-matrix through a normal distribution with a mean of the latent value and the corresponding standard deviation. Thus, the ith row of sub-matrix $\mathbf{X}$ is generated by randomly sampling p' values from $N(\mathbf{L}_i[2], Var(X))$, where p' is the number of features contained in sub-matrix $\mathbf{X}$. A similar process is repeated for the other sub-matrices to complete all components within $\mathcal{G}$ and $\mathcal{X}$ for each subject. After the simulation is complete for $\mathbf{Y}$, $\mathcal{G}$, and $\mathcal{X}$, the values are then centered as a final step. The signal strength may be controlled by calibrating the covariance structure to contain higher correlations amoungst the signal latent variables within $\mathcal{X}$ and $\mathcal{G}$.

Algorithm 1 describes the full details for the steps to execute the simulations performed in this manuscript. Additionally, the simulations may be performed through the simPathwaySystem function from the SuMOFil R package available at https://github.com/lorinmil/SuMOFil. The simPathwaySystem function involves many parameters to specify the strength of the network (*alpha*, *Rsq*, *rind*, *noiseSD*), number of features within the datasets (*nx*, *nxPrime*, *ns*, *ng*, *ngPrime*, *nh*, *ntranscriptNoise*, *ngeneNoise*), and sample size (*nsample*).

This manuscript performed simulations under 9 different simulation settings accounting for all possible

Algorithm 1 Network Simulation

Require: $alpha, Rsq, rind, noiseSD, nx, nxPrime, ns, ng, ngPrime, nh, ntranscriptNoise, ngeneNoise, nsample$

$\tau = 1$

$\beta^2_{Y|X.G} = \beta^2_{X|G} = \beta_{Y|S} = \beta_{Y|H} = alpha$

$tau = (1 - Rsq)/Rsq$

$\sigma_{Y|X.G.S.H} = tau + (alpha * tau)^2 + 2 * tau * alpha^2$

$\sigma_{X|G} = alpha * tau$

$\sigma_G = 1$

$\sigma_S = \sigma_{X|G} + \sigma_G * alpha$

$\sigma_H = \sigma_G$

$\sigma_{e_X} = \sigma_{e_G} = noiseSD^2$

Construct $\mathbf{\Sigma}_{9\times 9}$ using formulas from (2) and (3)

$tauInd = (1 - rind^2)/rind^2$

for $i = 1$ to $nsample$ **do**

  $\mathbf{L} \sim \mathbf{N}_9(\mathbf{0}, \mathbf{\Sigma})$

  $\mathbf{Y}[i] \sim N(\mathbf{L}[1], tauInd * \Sigma[1,1])$

  for $j = 1$ to nx **do**

    $\boldsymbol{\mathcal{X}}[i,j] \sim N(\mathbf{L}[2], tauInd * \Sigma[2,2])$

  end for

  for $j = nx + 1$ to $nx + ns$ **do**

    $\boldsymbol{\mathcal{X}}[i,j] \sim N(\mathbf{L}[4], tauInd * \Sigma[4,4])$

  end for

  for $j = nx + ns + 1$ to $nx + ns + nxPrime$ **do**

    $\boldsymbol{\mathcal{X}}[i,j] \sim N(\mathbf{L}[6], tauInd * \Sigma[6,6])$

  end for

  for $j = nx + ns + nxPrime + 1$ to $nx + ns + nxPrime + ntranscriptNoise$ **do**

    $\boldsymbol{\mathcal{X}}[i,j] \sim N(0, tauInd * \Sigma[8,8] * \Sigma[1,1])$

  end for

  for $j = 1$ to ng **do**

    $\boldsymbol{\mathcal{G}}[i,j] \sim N(\mathbf{L}[3], tauInd * \Sigma[3,3])$

  end for

  for $j = ng + 1$ to $ng + nh$ **do**

    $\boldsymbol{\mathcal{G}}[i,j] \sim N(\mathbf{L}[5], tauInd * \Sigma[5,5])$

  end for

  for $j = ng + nh + 1$ to $ng + nh + ngPrime$ **do**

    $\boldsymbol{\mathcal{G}}[i,j] \sim N(\mathbf{L}[7], tauInd * \Sigma[7,7])$

  end for

  for $j = ng + nh + ngPrime + 1$ to $ng + nh + ngPrime + ngeneNoise$ **do**

    $\boldsymbol{\mathcal{G}}[i,j] \sim N(0, tauInd * \Sigma[9,9] * \Sigma[1,1])$

  end for

end for

$\mathbf{Y} = \mathbf{Y} - \text{mean}(\mathbf{Y})$

$\boldsymbol{\mathcal{X}} = \boldsymbol{\mathcal{X}} - \text{mean}(\boldsymbol{\mathcal{X}})$

$\boldsymbol{\mathcal{G}} = \boldsymbol{\mathcal{G}} - \text{mean}(\boldsymbol{\mathcal{G}})$

return $\mathbf{Y}$, $\boldsymbol{\mathcal{X}}$, $\boldsymbol{\mathcal{G}}$

combinations of weak, moderate, and strong network signal strength and small, medium, and large sized datasets. The parameters used in this manuscript are summarized in Table A.

Table A: Simulation Parameter Settings.

**Signal Strength**			
**Parameter**	**Weak**	**Moderate**	**Strong**
alpha	0.35	0.35	0.35
Rsq	0.35	0.85	0.85
rind	0.35	0.35	0.85
noiseSD	0.5	1	2
**Number of Features**			
**Parameter**	**Small**	**Medium**	**Large**
nx	15	15	15
ns	50	50	50
nxPrime	100	100	100
ntranscriptNoise	5000	15000	25000
ng	10	10	10
nh	30	30	30
ngPrime	100	100	100
ngeneNoise	5000	10000	20000
