# Supplementary material for: SuMO-Fil: Supervised multi-omic filtering prior to performing network analysis
Source: PLoS One. 2021 Aug 3;16(8):e0255579. doi: 10.1371/journal.pone.0255579 (PMC8330944; doi:10.1371/journal.pone.0255579)
Supplement: S2 Appendix — (PDF) [file pone.0255579.s002.pdf]

S2 Appendix

Alternative Summary Measures for SuMO-Fil

Phase 2 of SuMO-Fil involves estimating a similarity matrix between datasets $\boldsymbol{\mathcal{G}}$ and $\boldsymbol{\mathcal{X}}$ and summing across the absolute values of the similarity for each feature to obtain a measure for filtering. Other summary measures of the absolute similarities such as medians were also considered as opposed to summation on a handful of simulations. Table A summarizes the average number of features filtered and the average number of network features filtering across 5 simulations for each of the simulation settings. Similar to summation, median summaries also do not remove network features under strong or moderate network signal strengths regarless of the number of features involved. However, under weak network signal there were some simulations that unnecessarily filtered network features. These results are consistent with summation used for summary functions and should be further explored.

Table A: Median Summary for Phase 2 in SuMO-Fil.

Simulation Settings			
Signal Strength	Total # Features	# Features Filtered	# Network Features Filtered
Weak	45305	$x = 3357$, $g = 2668$	$x = 0$, $g = 0$
Moderate	45305	$x = 3423$, $g = 2647$	$x = 0$, $g = 0$
Strong	45305	$x = 3796$, $g = 3115$	$x = 0$, $g = 0$
Weak	25305	$x = 2072$, $g = 1380$	$x = 0.4$, $g = 0$
Moderate	25305	$x = 2005$, $g = 1403$	$x = 0$, $g = 0$
Strong	25305	$x = 3030$, $g = 1991$	$x = 0$, $g = 0$
Weak	15305	$x = 729$, $g = 675$	$x = 0.2$, $g = 0.2$
Moderate	15305	$x = 731$, $g = 708$	$x = 0$, $g = 0$
Strong	15305	$x = 1127$, $g = 1101$	$x = 0$, $g = 0$

SuMO-Fil was performed using median as opposed to summation for the second phase. This displays the average results based on 5 simulations under each simulation setting. Ideally, no network features should be removed while still reducing the total number of features in the datasets.
